# Involvement of urokinase receptor in the cross-talk between human hematopoietic stem cells and bone marrow microenvironment

**DOI:** 10.18632/oncotarget.11115

**Published:** 2016-08-08

**Authors:** Carmine Selleri, Nunzia Montuori, Annamaria Salvati, Bianca Serio, Ada Pesapane, Patrizia Ricci, Anna Gorrasi, Anna Li Santi, Gunilla Hoyer-Hansen, Pia Ragno

**Affiliations:** ^1^ Department of Medicine and Surgery, University of Salerno, Salerno, Italy; ^2^ Department of Translational Medical Sciences, “Federico II” University, Naples, Italy; ^3^ Department of Chemistry and Biology, University of Salerno, Salerno, Italy; ^4^ The Finsen Laboratory, Rigshospitalet, Copenhagen, Denmark

**Keywords:** urokinase receptor, uPAR, hematopoietic stem cells, bone marrow microenvironment, leukemia

## Abstract

Hematopoietic stem cells (HSCs) reside in bone marrow (BM) and can be induced to mobilize into the circulation for transplantation. Homing and lodgement into BM of transplanted HSCs are the first critical steps in their engraftment and involve multiple interactions between HSCs and the BM microenvironment.

uPAR is a three domain receptor (DIDIIDIII) which binds urokinase, vitronectin, integrins. uPAR can be cleaved and shed from the cell surface generating full-length and cleaved soluble forms (suPAR and DIIDIII-suPAR). DIIDIII-suPAR can bind fMLF receptors through the SRSRY sequence (residues 88-92).

We previously reported the involvement of soluble uPAR in HSC mobilization. We now investigate its possible role in HSC homing and engraftment.

We show similar levels of circulating full-length suPAR in healthy donors and in acute myeloid leukemia (AML) patients before and after the pre-transplant conditioning regimen. By contrast, levels of circulating DIIDIII-suPAR in AML patients are higher as compared to controls and significantly decrease after the conditioning.

We found that suPAR and uPAR_84-95_, a uPAR-derived peptide which mimics active DIIDIII-suPAR, induce a significant increase in Long Term Culture (LTC)-Initiating Cells (ICs) and in the release of clonogenic progenitors from LTCs of CD34^+^ HSCs. Further, suPAR increases adhesion and survival of CD34^+^ KG1 AML cells, whereas uPAR_84-95_ increases their proliferation.

Thus, circulating DIIDIII-suPAR, strongly increased in HSC mobilization, is indeed down-regulated by pre-transplant conditioning, probably to favour HSC homing. BM full-length suPAR and DIIDIII-suPAR may be involved in HSC lodgement within the BM by contributing to a suitable microenvironment.

## INTRODUCTION

Hematopoietic stem cells (HSCs) are clonogenic cells capable of self-renewal and multilineage differentiation. The expression of the CD34 antigen and lineage negativity are commonly used in clinics to identify human HSCs. The majority of HSCs resides in the bone marrow (BM), which provides them with a suitable microenvironment, regulating their proliferation/survival and differentiation [1–2]. HSCs are retained in the BM mainly by their adhesive interactions with the cellular and matrix components of the stroma and by interaction with specific BM chemokines, in particular the stromal-derived factor 1 (SDF1), through the CXCR4 receptor [1–2]. HSCs can be forced to mobilize into the circulation [3]. Currently, mobilized peripheral blood (PB) HSCs represent the major source of stem cells for autologous stem cell transplantion (SCT); they are also increasingly used in allogeneic SCT, because of the relative ease of collection, the higher yield of stem cells and the shorter time to engraftment. The most common mobilizer is the granulocyte-colony stimulating factor (G-CSF) [3–4].

Previously, we reported the involvement of the urokinase receptor (uPAR) in HSC mobilization [5–6]. uPAR consists of three homologous domains (DI, DII, DIII) anchored to the cell surface by a glycosyl-phosphatidylinositol (GPI) tail and concentrates the proteolytic activity of its ligand, the serine protease urokinase (uPA), on the cell membrane, to promote plasminogen activation and focused extracellular matrix (ECM) degradation [7]. However, uPAR is endowed with proteolysis-independent activities; in fact, it binds vitronectin (VN), a component abundant in tumor-associated ECM, interacts with various integrins, regulating their activity, mediates uPA-dependent cell migration and is required for chemotaxis induced by fMet-Leu-Phe (fMLF), a potent leukocyte chemoattractant [8]. uPAR can be cleaved, thus generating cell-surface truncated forms lacking the N-terminal domain (DIIDIII-uPAR) [9]. Both full-length and cleaved forms can be shed from the cell surface (suPAR and DIIDIII-suPAR, respectively). DIIDIII-suPAR exposing residues 88-92 (SRSRY), is a ligand for the receptors of fMLF (fMLF-Rs) and induces migration of various cell types [10–11]. The cleaved form of soluble uPAR also regulates the activity of inflammatory chemokine receptors, such as MCP-1 and RANTES receptors, through fMLF-R activation [12].

We previously found that G-CSF-mobilized human HSCs were uPAR-negative; however, G-CSF administration to HSC healthy donors up-regulated uPAR expression in circulating myeloid precursors and monocytic cells, and increased suPAR and DIIDIII-suPAR levels in sera. DIIDIII-suPAR likely contributed to HSC mobilization by inducing migration of BM-HSCs into the circulation [5–6]. In mice, the membrane-anchored uPAR marks a subset of BM hematopoietic stem/progenitor cells (HSPCs) and promotes the preservation of the size of this pool of cells. During G-CSF mobilization in mouse, uPAR is released from the HPSC surface, likely by plasmin activation; uPAR loss impairs HSPC homing and engraftment to the BM microenvironment [13].

We now analyze the levels of circulating soluble uPAR forms before and after the myeloablative conditioning regimen preceding HSC transplantation, to identify variations suggesting a possible involvement also in HSC homing and engraftment to BM. Then, we explored a potential uPAR role in the cross-talk between HSCs and BM. We characterized the presence of the different forms of uPAR and of its extracellular ligands, uPA and VN, in BM stroma; then, we examined the effects of soluble uPAR forms in long term cultures of CD34^+^ HSCs and on the KG1 cell line, as a model for CD34^+^ hematopoietic cells [14].

## RESULTS

### The level of circulating cleaved forms of soluble uPAR are lowered by chemotherapy-based pre-transplant conditioning

Acute myeloid leukemia (AML) patients, before HSC transplantation, are subjected to a myeloablative conditioning regimen aimed to eradicate the underlying hematologic malignancy and to suppress the host immune system. First of all, we evaluated whether the pre-transplant treatment induced variations in the levels of circulating full-length and cleaved suPAR forms.

**Figure 1:**
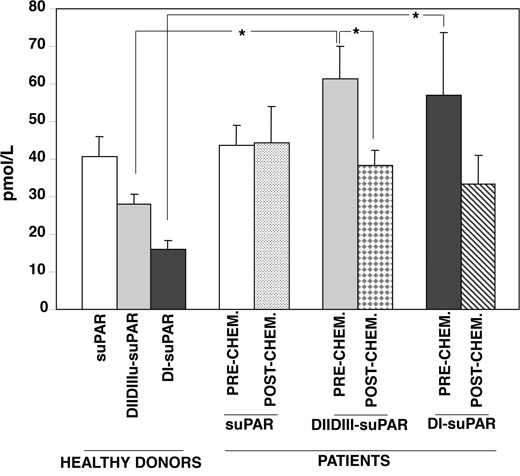
Circulating cleaved forms of suPAR are lowered by chemotherapy-based conditioning regimen Circulating forms of soluble uPAR forms were measured in healthy donors (n=4) and in AML patients (n=5) before and after the pre-transplantation chemotherapy-based conditioning regimen, using a time-resolved fluorescence immunoassay [31]. This technique allows to quantitate full-length suPAR and its cleavage products, DIIDIII-suPAR and DI-suPAR. (*) p≤0.05, as determined by the Student's *t* test.

Circulating uPAR forms were measured in healthy donors and in patients using a time-resolved fluorescence immunoassay (Figure 1). This technique allows to quantitate full-length suPAR and its cleavage products, DIIDIII-suPAR and DI-suPAR.

Different levels of the three suPAR forms were detected in plasma from four healthy donors. The levels of circulating full-length suPAR were similar in healthy donors and AML patients, and were not influenced by the conditioning regimen. By contrast, the levels of suPAR cleavage products (DIIDIII-suPAR and DI-suPAR) were significantly higher in AML patients as compared to healthy donors; the pre-transplant chemotherapy treatment lowered both suPAR forms, although only the DIIDIII-suPAR decreased in a significant manner.

These results suggest that AML blasts mainly release DIIDIII-suPAR; further, the pre-transplant treatment decreases circulating DIIDIII-suPAR level, according with the previously observed increase of DIIDIII-suPAR during HSC mobilization [5].

### Bone marrow stroma cells express uPAR and its ligands

We then investigated uPAR expression in BM stroma. Human CD34^+^ HSCs residing in the BM do not express uPAR [5, 15]. However, uPAR and its extracellular ligands may be expressed by BM stroma cells and contribute to a suitable microenvironment, thus favouring HSC lodgement and engraftment to BM.

**Figure 2:**
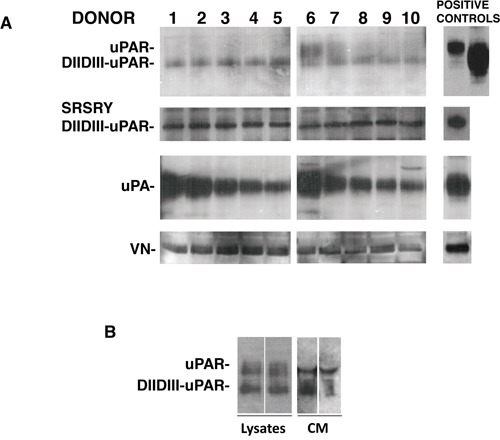
Bone marrow stroma cells express uPAR and its ligands Bone marrow stroma cells from ten healthy donors were cultured in long-term stem cell medium and then lysed. 50 μg of cell lysate was analyzed by Western blot with a monoclonal antibody able to recognize both full-length and cleaved uPAR (first inset), a polyclonal antibody directed to the uPAR_84–95_ region (second inset), and specific antibodies for uPA and vitronectin (VN) **(Panel A).** Bone marrow stroma cells from two further healthy donors were cultured at subconfluence and incubated with serum-free culture medium additioned with 1 mg/ml BSA for 3 days at 37°C, 5% CO_2_. Then, TCA precipitated incubation media and 50 μg of cell lysates were analyzed by Western blot with the uPAR specific monoclonal antibody **(Panel B).**

The presence of the different uPAR forms and of uPAR ligands was evaluated in cultures of human BM stroma cells obtained from ten healthy donors (Figure 2A). Western blot with a monoclonal antibody able to recognize both full-length and cleaved uPAR, showed expression of full-length uPAR in some analyzed samples and expression of cleaved uPAR in all analyzed samples. Interestingly, a polyclonal antibody directed to the uPAR_84–95_ region, which contains the binding sequence for fMLF receptors (residues 88–92), recognized the uPAR cleaved form in all samples, indicating the exposition of this active region. Both uPAR extracellular ligands, uPA and VN, were expressed by BM stroma cells.

Membrane-anchored uPAR forms can be easily shed from the cell surface by specific phospholipases [7]. In fact, both full-length and cleaved suPAR were released in cultures of BM cells (Figure 2B).

These results indicate that all the different forms of uPAR and both uPAR ligands are expressed in BM stroma.

### Soluble uPAR forms increase the number of LTC-ICs and the release of clonogenic progenitors in long-term cultures of PB-CD34^+^ cells

**Figure 3:**
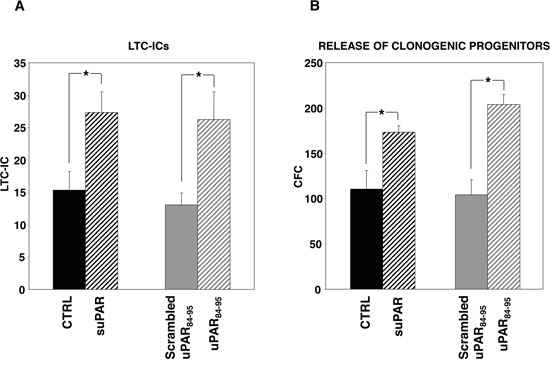
Soluble uPAR forms increase the number of LTC-ICs and clonogenic progenitors in long-term cultures of PB-CD34^+^ cells CD34^+^ cells purified from PB of four donors were plated on BM-derived stroma, allowed to adhere, and cultured for 5 weeks with or without suPAR, uPAR_84-95_ peptide or its scrambled version. At the end of the fifth week, adherent cells were harvested and cultured in methylcellulose for LTC-ICs calculation **(Panel A).** Output of clonogenic progenitors in culture medium was assayed by replating non-adherent cells in methylcellulose for short term cultures; myeloid and erythroid colonies were counted as colony forming cells (CFCs) **(Panel B).** (*) p≤0.05, as determined by the Student's *t* test.

We then evaluated the potential roles of soluble uPAR in the cross-talk between HSCs and the BM microenvironment. The effect of full-length suPAR and of the DIIDIII-suPAR-derived peptide, uPAR_84-95_, on clonogenic progenitors, was studied in long-term cultures (LTCs) of PB-CD34^+^ cells. uPAR_84-95_ covers the uPAR region corresponding to residues 84-95, able to bind and activate fMLF-Rs, thus mimicking the DIIDIII-suPAR form which exposes this region [10, 16–17]. LTCs, at present, represent the best surrogate *in vitro* of BM hematopoiesis *in vivo*, because they mimic the complex intercellular interactions between the stromal microenvironment and stem cells concentrated in niches of hematopoietic activity. Highly purified PB-CD34^+^ cells from four donors were grown on irradiated BM-derived stroma cells, in the presence or in the absence of suPAR, uPAR_84-95_ peptide or its scrambled version. Then, adherent cells were harvested and cultured in methylcellulose for LTC-ICs calculation. The output of clonogenic progenitors in culture medium of LTCs was also assayed; myeloid and erythroid colonies were counted as colony forming cells (CFCs). Both suPAR and uPAR_84-95_ peptide significantly increased LTC-ICs number as compared to the respective controls (Figure 3A). Both stimuli increased also the release of clonogenic progenitors (Figure 3B), expanding previous results showing this effect only for the uPAR_84-95_ peptide in LTCs of BM-CD34^+^ cells [5].

These results indicate that both forms of soluble uPAR increase the absolute number of LTC-ICs and, possibly as a consequence, the release of clonogenic progenitors from BM stroma.

### Full-length suPAR increases adhesion of CD34^+^ KG1 cells to fibronectin

suPAR or uPAR_84-95_ peptide may increase the number of LTC-ICs in PB-CD34^+^ LTCs by promoting their adhesion to BM stroma. To test this possibility we evaluated the effects of both stimuli on the human CD34^+^ KG1 cell line [14].

**Figure 4:**
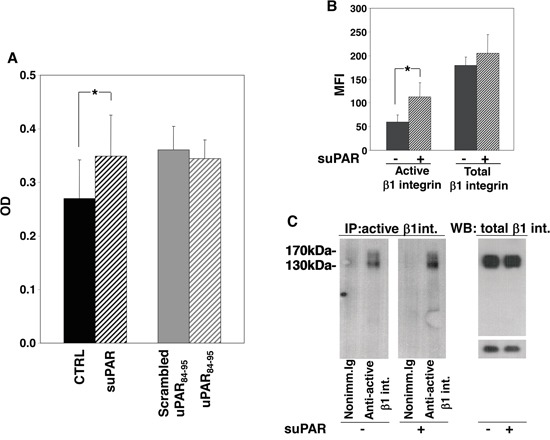
Full-length suPAR increases adhesion of KG1 cells to fibronectin **Panel A:** KG1 cells were resuspended in 1mg/ml BSA-RPMI at a density of 1×10^6^ cells/ml, pre-incubated with or without suPAR (20 nM), uPAR_84-95_ or scrambled peptide (10 nM) for 30′ at 37°C and plated on plastic-bound fibronectin for 16 h at 4°C. Attached cells were fixed with 3% PFA and stained with crystal violet; stain was eluted and its absorbance at 540 nm was measured by a spectrophotometer. (*) p≤0.05, as determined by the Student's *t* test. **Panel B:** suPAR-treated or untreated KG1 cells were fixed with PFA, incubated with antibodies against total or active β1 integrin or with nonimmune mouse Ig and, then, with FITC anti-mouse IgG. Cells were finally analyzed by flow cytometry using a FACScan (MFI: Mean Fluorescence Intensity). (*) p≤0.05, as determined by the Student's *t* test. **Panel C:** Cell surface antigens of suPAR-treated or untreated KG1 cells were biotinylated with EZ-link sulfo-NHS-LC-biotin. Cells were lysed; 50 μg of cell lysates were analyzed by Western blot with an antibody against total β1 integrin, 400 μg of cell lysates were incubated with an antibody against active β1 integrin or nonimmune mouse Ig. Immunocomplexes were then precipitated by protein A-Sepharose, eluted and analyzed by Western blot with horseradish peroxidase-conjugated streptavidin.

KG1 cells were incubated with or without full-length suPAR, uPAR_84-95_ peptide or its scrambled version. Then, cells were plated on plastic-bound FN or VN. Cells did not adhere to VN (not shown) whereas efficiently adhered to FN, which, indeed, is largely present in the BM stroma. Only full-length suPAR significantly increased KG1 cell adhesion to FN, whereas the uPAR_84-95_ peptide did not exert any effect as compared to its scrambled control (Figure 4A).

Increased adhesion of suPAR-treated KG1 cells to FN may be due to increased levels of specific integrins or to the increase of their activation. Therefore, we evaluated the levels and the activation state of β1 integrins in suPAR-treated or untreated KG1 cells. We focused on β1 integrins since they generally mediate cell adhesion to FN, in particular VLA-4 (α4β1), and VLA-5 (α5β1), which are expressed on hematopoietic progenitors and on KG1 cells [14]. We investigated the activation state of surface β1 integrin by flow cytometry, showing that suPAR increased the level of active β1 integrin without affecting its expression (Figure 4B). Similar results were obtained by immunoprecipitation of active β1 integrin (Figure 4C).

These results suggest that suPAR may act in LTC-IC assays by promoting CD34^+^ cell adhesion to the BM stroma.

### uPAR_84-95_ increases CD34^+^ KG1 cell proliferation

**Figure 5:**
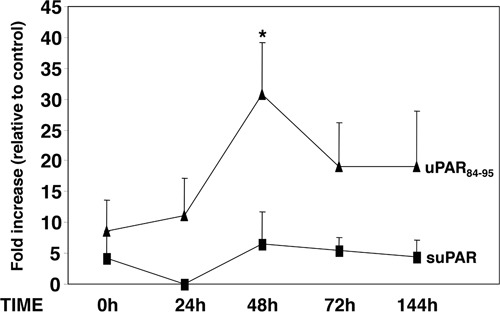
uPAR_84-95_ increases KG1 cell proliferation KG1 cells were serum-starved overnight, plated on plastic-bound fibronectin (FN) and cultured with or without full-length suPAR (20nM) (■), uPAR_84-95_ peptide (10 nM) or it scrambled version (10 nM) (▲) for 0, 24, 48, 72 hours or 6 days (144h). At indicated times, 20μl/well of CellTiter 96 AQueous One Solution Reagent was added. After incubation for 4 h at 37°C, 5% CO_2_, the absorbance was determined by an ELISA reader (Bio-Rad) at a wavelength of 490 nm. Fold increase represents the ratio between values obtained by treated cells and values obtained by the corresponding control at each time. (*) p≤0.05, as determined by the Student's *t* test, compared to time 0.

An increased proliferation may also contribute to the observed increase in the number of LTC-ICs in PB-CD34^+^ LTCs; indeed, the increased pro-adhesive effect of suPAR, shown in the previous paragraph, may favour also cell proliferation. To test this possibility we evaluated the effects of both stimuli on KG1 cell proliferation. KG1 cells were cultured on FN with or without full-length suPAR, uPAR_84-95_ peptide or its scrambled version for 0, 24, 48, 72 hours or 6 days (144h). uPAR_84-95_ significantly increased KG1 cell proliferation after 48 h, whereas no effect was observed with suPAR treatment (Figure 5).

Thus, only uPAR_84-95_ may directly affect proliferation of CD34^+^ cells, even if it does not exert any effect on their adhesion to FN.

### Full-length suPAR protects CD34^+^ KG1 cells from apoptosis

**Figure 6:**
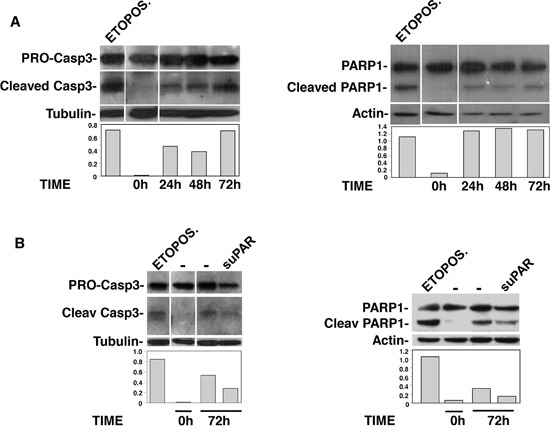
Full-length suPAR protects KG1 cells from apoptosis KG1 cells were serum starved for indicated times, in the absence **(Panel A)** or in the presence of full-length suPAR or diluent **(Panel B).** Cells were then lysed and analyzed by Western blot with antibodies directed to full-length/cleaved Caspase3 or Parp1; not starved KG1 cells and KG1 cells treated with etoposide were used as negative and positive apoptosis control, respectively. The graphs show the O.D. obtained by densitometric scanning of cleaved caspase/Parp1 bands normalized to the O.D. of corresponding tubulin/actin bands.

suPAR or uPAR_84-95_ peptide may also act in PB-CD34^+^ LTCs by protecting HSCs from apoptosis. Thus, apoptosis of KG1, induced by serum deprivation, was evaluated in the presence or absence of full-length suPAR, uPAR_84-95_ peptide or its scrambled version. Apoptosis was examined by evaluating cleavage of Caspase3 or PARP1. Not starved KG1 cells and KG1 cells treated with etoposide were used as negative and positive apoptosis control, respectively. First, KG1 cells were serum starved for different times to evaluate their survival. Western blot analysis showed that Caspase3 and PARP1 cleavage already occurred after 24 h, but Caspase3 cleavage was more evident after 72 h of serum starvation (Figure 6A). Thus, suPAR and uPAR_84-95_ effects on KG1 cell apoptosis were examined after 72 h of serum deprivation. Indeed, full-length suPAR was able to reduce Caspase3 and PARP1 cleavage as compared to the control (Figure 6B), suggesting that this uPAR form may protect KG1 cells from apoptosis; uPAR_84-95_ did not seem to affect Caspase3 and PARP1 cleavage as compared to the scrambled peptide (not shown).

These results suggest that full-length suPAR may contribute to the increase of LTC-ICs also by supporting their survival.

## DISCUSSION

In BM niches, HSCs warrant blood cell homeostasis and the maintenance of a stable pool of undifferentiated cells. In fact, HSCs possess the capacity for self-renewal and multilineage differentiation. Microenvironment contributes to maintain a dynamic balance between these critical activities [18].

Treatments leading to a better chance of long-term remission for malignant blood disorders as leukemia can involve myeloablation followed by transplantation of matched donor HSCs. Donor HSCs can be forced to “mobilize” into the blood to allow their harvesting for transplantation. In the recipient, transplanted HSCs must first “home” to the BM, then localize and anchor within the BM, a process known as lodgement. Homing and lodgement of transplanted HSCs are the first critical steps in engraftment and involve multiple interactions between HSCs and the BM microenvironment. Indeed, these interactions dictate the clinical outcome of the transplantation [19].

We previously reported the involvement of suPAR in HSC mobilization. In fact, we observed that G-CSF increased the levels of circulating soluble forms of uPAR, both the full-length and the cleaved (DIIDIII) form [5]. On the basis of *in vitro* and *in vivo* experiments, we hypothesized a role for the circulating cleaved suPAR, which may contribute to HSC mobilization by directly chemoattracting HSCs into the circulation.

On the other hand, in mice, the membrane-anchored uPAR, expressed by a subset of BM-HSPCs, contributes to the maintenance of this pool of cells in BM [13]. In addition, it has been reported that supernatants of leukapheresis products (SLPs) of patients mobilized with G-CSF, or the various components of SLPs, which include the full-length soluble uPAR, increase the chemotactic response of HSPCs to SDF-1, even if they are not able to directly chemoattract HSCs [20–21]. These observations supported the hypothesis that soluble uPAR may be involved also in HSC homing/lodgement to BM.

On these basis, we firstly evaluated the levels of circulating full-length suPAR, DIIDIII-suPAR or the released DI domain in healthy donors and in AML patients, before and after the pre-transplant conditioning. Unexpectedly, we found similar levels of full-length suPAR in donors and in patients before and after the conditioning, while both fragments of suPAR were significantly higher in patients as compared to healthy donors. Circulating suPAR fragments strongly decreased in patients after the conditioning, at the point of most pronounced aplasia, suggesting that main suPAR forms released by AML blasts are probably the cleaved suPAR forms.

Indeed, increased suPAR levels have been reported in plasma from AML patients without distinction among the various forms [22], thus it is possible that the increase is due to the specific increase of suPAR fragments. Recently, suPAR was measured by ELISA in plasma daily taken from a mixed group consisting in ALL, few AML and hematologic patients, during the pre-transplant conditioning with antithymocyte globulin. suPAR levels before the start of the conditioning were only slightly elevated as compared to those of healthy controls; during the conditioning there was a significant increase in suPAR levels only after the first day of treatment, hereafter, suPAR levels generally declined [23]. Thus, our results are in agreement with previous observations and extend them by showing which forms of suPAR are modulated in AML and during pre-transplant conditioning. Increased levels of cleaved suPAR in AML patients, as compared to control healthy donors, may better reflect the number of leukemic blasts respect to intact suPAR, as already demonstrated in mouse breast cancer [24].

These results are also in agreement with our previous observation on the mobilizing effects of cleaved DIIDIII-suPAR [5–6], which is indeed reduced by pre-transplant conditioning and thus cannot counteract homing and lodging of HSCs in BM.

uPAR and/or its extracellular ligands may be expressed in BM stroma cells, thus also favouring engraftment to BM. To elucidate this point, we first assessed the presence of the different uPAR forms in BM stroma. Since membrane-anchored uPAR forms can be easily shed from the cell surface by specific phospholipases [7] and act on neighbouring HSCs, we explored, by LTCs, the effects of full-length suPAR and of a uPAR-derived peptide, uPAR_84-95_, covering the active region of DIIDIII-suPAR, on HSCs obtained from PB of healthy donors. The addition of both suPAR forms induced a significant increase in the absolute number of LTC-ICs and in the release of clonogenic progenitors, suggesting that they could contribute to the BM engraftment by both promoting HSCs proliferation/differentiation and self-renewal.

Since the increased number of LTC-ICs and clonogenic progenitors could be due to various events, we investigated the effects of both forms of suPAR on adhesion, proliferation or survival of CD34^+^ KG1 cells, as a model of CD34^+^ hematopoietic cells [14]. Interestingly, full-length suPAR induced an increase in adhesion and survival of KG1 cells, likely by regulating integrin-dependent signalling, as previously shown in different cell types [25–26]. uPAR_84-95_ did not seem to exert any effect on adhesion and survival of KG1 cells, however, it increased their proliferation, unlike full-length suPAR. Since cleaved uPAR lacking DI does not associate with integrins but is a ligand of fMLF-Rs, such as its derived peptide, uPAR_84-95_, proliferative effect could be due to the stimulation of fMLF-Rs expressed by KG1 cells [5], which have been reported to be able to activate proliferative signals beside migratory pathways [27–28].

In conclusion, our results show that circulating DIIDIII-suPAR, strongly involved in HSC mobilization, is indeed down-regulated by pre-transplant conditioning. Interestingly, BM full-length suPAR and DIIDIII-suPAR could be involved in the maintenance of HSCs within the BM by contributing to a suitable microenvironment for HSC engraftment since they are present in the BM stroma and exert various and significant effects on CD34^+^ cells.

## MATERIALS AND METHODS

### Reagents

Anti-uPA polyclonal antibody was from American Diagnostica (Greenwich, CT); suPAR from R&D system (Minneapolis, MN). The rabbit antibody, recognizing the SRSRY sequence of uPAR [29], and uPAR_84-95_ (AVTYSRSRYLEC) and scrambled (TLVEYYSRASCR) peptides were purchased from PRIMM (Milan, Italy). Rabbit anti-actin, mouse anti-tubulin antibodies, hydrocortisone sodium hemisuccinate, the protease inhibitor cocktail were from Sigma-Aldrich (St. Louis, MO). Polyclonal anti-caspase3 and anti-VN antibodies, the monoclonal anti-PARP1 and anti-β1 integrin antibodies were purchased from Santa Cruz (Santa Cruz, CA); horseradish peroxidase-conjugated secondary antibodies from Bio-Rad (Hercules, CA) and FITC anti-mouse IgG from Jackson Lab. (West Grove, PA). EZ-link sulfo-NHS-LC-biotin was from Thermo Scientific (Rockford, USA) and Streptavidin from GE-Healthcare (UK). ECL detection kit was from Amersham International (Amersham, England), Polyvinylidene fluoride (PVDF) filters and anti-active β1 integrin (clone 12G10) antibody from Millipore (Windsor, MA). Lymphocyte separation medium was from Life Technologies (Gaithersburg, MD); anti-CD3 Abs and IgG-conjugated magnetic beads for immunedepletion were from Life Technologies (Carlsbad, CA). Fluorescein isothiocyanate (FITC)-, peridinin chlorophyll (PerCP)- and phycoerythrin (PE)-labeled monoclonal antibodies were purchased from Becton Dickinson (Mountain View, CA, USA). Long-term stem cell medium and methylcellulose were from Stem Cell Technologies (Vancouver, Canada). IL-3, G-CSF, GM-CSF, SCF and EPO were from Amgen (Thousand Oaks, CA, USA). Fibronectin was from Roche (Indianapolis, USA). The kit for Proliferation Assay was from Promega (Madison, WI, USA).

### Sample collection

Heparinized blood samples were obtained after written informed consent (according to the procedures outlined by the ethical committee of our institution) from 5 AML patients [mean age 39±16 years; one of them with a favorable cytogenetic risk due to the presence of inv(16), two with an intermediate risk showing a normal karyotype and the last 2 with an unfavorable risk due to the presence of a complex karyotype and t(9;22), respectively] before and after the pretransplant conditioning with chemotherapy and from four healthy donors. Plasma samples were prepared by two successive centrifugations to achieve complete platelet removal and were stored at −80°C. All patients underwent conditioning with the BU-CY2 regimen (busulfan, 16 mg/kg; cyclophosphamide, 120 mg/kg) and received cyclosporin A (CsA) (1 mg/kg·day by continuous i.v. infusion from day −1 to day + 20 and then 8 mg/kg·day orally) plus short-course methotrexate (10 mg/m^2^ on days +1, +3, and +6) as prophylaxis for GVHD. All patients obtained full donor engraftment after a mean of 47±15 days from transplant. Heparinized bone marrow specimens for BM stroma analysis were obtained by aspiration from the posterior iliac crest from 12 healthy young donors at the time of allogeneic marrow donation, after informed consent.

### Cell cultures

For marrow stromal layer, 5×10^6^/ml BM mononuclear cells (MNCs) were resuspended in a culture medium that consisted of long-term stem cell medium (Myelocult; StemCell Technologies) supplemented with 1×10^−6^mol/liter hydrocortisone sodium hemisuccinate (Sigma), plated into 25-cm^2^ tissue culture flasks and incubated in a humidified atmosphere (37°C, 5% CO_2_). On a weekly basis, the stromal layer cultures were fed by complete replacement of the medium and analyzed for stromal confluence after 4-5 weeks.

CD34^+^ KG1 acute myelogenous leukemia cells were cultured in RPMI 1640 supplemented with 10% heat-inactivated fetal bovine serum (FBS).

### CD34^+^ hematopoietic stem cell isolation

Peripheral blood (PB) MNCs were isolated by density gradient centrifugation using lymphocyte separation medium. After washing with phosphate-buffered saline (PBS) supplemented with 1% BSA, CD34^+^ cells were highly purified by MiniMacs high-gradient magnetic separation columns (Miltenyi Biotec, Auburn, CA) according to the manufacturer's instructions. Briefly, MNCs were incubated with a hapten-conjugated anti-CD34^+^ mAb in the presence of an Fc receptor blocking reagent. These cells were subsequently incubated with MACS microbeads conjugated to an anti-hapten antibody and purified using a high-gradient magnetic separation column (Miltenyi Biotec). Positive cells were discharged from the column after removal from the magnetic field. CD34^+^ cells were enriched to 95% purity by 2 sequential selections through the magnetic cell separator. Purity of the positively selected CD34^+^ cells was evaluated by flow cytometry. Purified CD34^+^ cells were resuspended in RPMI medium supplemented with 1% BSA.

### LTC-IC assay

For long-term cultures (LTCs), highly purified PB-CD34^+^ cells were plated on irradiated (15 Gy of 250kV rays) allogeneic bone marrow stromal cells (3×10^4^/cm^2^) at a cell density of 1×10^5^ per well. Culture medium consisted of long-term stem cell medium supplemented with 1×10^−6^ M hydrocortisone sodium hemisuccinate. LTCs were treated with or without 1μg/ml suPAR, uPAR_84-95_ peptide or its scrambled version, incubated at 37°C for 3 days and then switched at 33°C for 5 weeks. Stimuli were weekly re-added to LTC culture medium. Weekly removed non-adherent cells were washed and used in replating experiment to measure the output of clonogenic progenitors. At the end of 5 weeks, also stroma-adherent cells were harvested by trypsinization, washed three times and assayed for their content of clonogenic progenitors, as non-adherent cells. Each experimental procedure was performed in duplicate.

Clonogenic progenitors were measured in methylcellulose, as previously described [30]. Briefly, cells were plated in 1 ml medium per dish (35 mm dishes) in the presence of the following growth factor cocktail: 10 ng/ml IL-3, 50 ng/ml G-CSF, 50 ng/ml GM-CSF, 20 ng/ml SCF, and 2 U/ml EPO. Myeloid and erythroid colonies were counted as colony forming cells (CFCs) after 14 days incubation at 5% CO_2_.

### Adhesion assay

96-well flat-bottom microtiter plates were coated with 10 μg/ml vitronectin (VN) or fibronectin (FN) or with 1% heat-denatured BSA-PBS as a negative control, and incubated overnight at 4°C. The plates were then blocked 1 h at room temperature with 1% heat-denatured BSA in PBS. KG1 cells were resuspended in 0.1% BSA-RPMI at a density of 1×10^6^ cells/ml, pre-incubated with or without suPAR (20 nM), uPAR_84-95_ or scrambled peptide (10 nM) for 30′ at 37°C, plated in previously coated plates and incubated for 1 h at 37°C, 5% CO_2_. Then, wells were washed and attached cells were fixed with 3% paraformaldehyde in PBS for 10 min and incubated with 2% methanol for 10 min. The cells were finally stained for 10 min with 0.5% crystal violet in 20% methanol. Stain was eluted by 0.1 M sodium citrate in 50% ethanol, pH 4.2, and the absorbance at 540 nm was measured by a spectrophotometer.

### Western blot analysis

Cells were lysed in 1% Triton X-100 in the presence of protease inhibitors. The protein content of cell lysates was measured by a colorimetric assay (BIORAD); cell lysates were electrophoresed in SDS-PAGE (10% for uPAR and PARP1 detection, 12% for Caspase3 detection) and transferred onto a PVDF filter. The membrane was blocked with 5% milk and probed with 1μg/ml of specific antibody. Finally, washed filters were incubated with horseradish peroxidase-conjugated secondary antibodies and bands detected by ECL.

### Cell surface biotinylation and immunoprecipitation

suPAR-treated or untreated KG1 cells were washed in PBS and incubated with EZ-link sulfo-NHS-LC-biotin 1 mg/ml in PBS for 30′ at room temperature, with constant gentle agitation. The supernatant was then removed and the reaction stopped by the addition of 100 mM glycine. Finally, cells were washed and lysed in RIPA buffer.

Biotinylated samples were incubated 2 h at 4°C with 30 μg/ml of anti-β1 integrin monoclonal antibodies or nonimmune mouse immunoglobulins. Immunocomplexes were then precipitated by protein A-Sepharose, eluted and analyzed by Western blot with horseradish peroxidase-conjugated streptavidin diluted 1:3000.

### Flow cytometry analysis

suPAR-treated or untreated KG1 cells were washed and fixed with 4% PFA for 30′ on ice; fixed cells were washed and incubated in blocking solution (5% BSA in PBS) for 30′ on ice. Cells were then incubated with 10 μg/ml anti-β1 integrin antibodies or nonimmune mouse immunoglobulins for 3 h on ice, washed and incubated with fluorescein isothiocyanate-labeled anti-mouse IgG in 5% BSA for 1 h on ice. Finally, the cells were washed and analyzed by flow cytometry using a FACScan (Becton Dickinson, San Josè, CA).

### Cell proliferation assay

KG1 cells were incubated in 0.5% FBS-RPMI for 16 h at 37°C, 5% CO_2_. Then, 1×10^5^/ml cells were plated in FN- or VN-coated 96-well plates (10 μg/ml) and incubated with suPAR (20 nM), uPAR_84-95_ (10 nM), scrambled peptide (10 nM) or medium. At indicated times, 20μl/well of CellTiter 96 AQueous One Solution Reagent was added. After incubation for 4 h at 37°C, 5% CO_2_, the absorbance was determined by an ELISA reader (Bio-Rad) at a wavelength of 490 nm.

### Measurement of soluble uPAR forms

The concentration of the three soluble uPAR forms was determined using three time-resolved fluorescence assays (TR-FIA): TR-FIA1 measuring full-length suPAR, TR-FIA2 measuring full length suPAR + DIIDIII-suPAR, and TR-FIA4 measuring DI-suPAR [31]. Fluorescence was measured using a FluoStar Galaxy fluorometer (BMG LabTechnologies) with excitation at 405 nm and emission at 615 nm.

### Statistical analysis

Differences between groups were evaluated by the Student's t test using PRISM software (GraphPad, San Diego, CA). *P≤*0.05 was considered statistically significant.
